# Active Vibration Control and Parameter Optimization of Genetic Algorithm for Partially Damped Composites Beams

**DOI:** 10.3390/biomimetics9100584

**Published:** 2024-09-25

**Authors:** Zhicheng Huang, Yang Cheng, Xingguo Wang, Nanxing Wu

**Affiliations:** College of Mechanical and Electronic Engineering, Jingdezhen Ceramic University, Jingdezhen 333001, China; 15270811356@163.com (Y.C.); wangxingguo@jci.edu.cn (X.W.); wunanxing@jci.edu.cn (N.W.)

**Keywords:** composites beam, piezoelectric actuator, finite element analysis, vibration control, Kalman filter, linear–quadratic–Gaussian control, genetic algorithm

## Abstract

The paper partially covered Active Constrained Layer Damping (ACLD) cantilever beams’ dynamic modeling, active vibration control, and parameter optimization techniques as the main topic of this research. The dynamic model of the viscoelastic sandwich beam is created by merging the finite element approach with the Golla Hughes McTavish (GHM) model. The governing equation is constructed based on Hamilton’s principle. After the joint reduction of physical space and state space, the model is modified to comply with the demands of active control. The control parameters are optimized based on the Kalman filter and genetic algorithm. The effect of various ACLD coverage architectures and excitation signals on the system’s vibration is investigated. According to the research, the genetic algorithm’s optimization iteration can quickly find the best solution while achieving accurate model tracking, increasing the effectiveness and precision of active control. The Kalman filter can effectively suppress the impact of vibration and noise exposure to random excitation on the system.

## 1. Introduction

The significance of vibration on the longevity and safety of mechanical systems has increased in recent years. The limitations of conventional vibration reduction techniques have raised awareness about the need for structural vibration reduction. An increasing number of researchers are conducting in-depth studies on vibration reduction structures, motivated by ACLD’s innovative solution that combines traditional viscoelastic damping with piezoelectric materials. When combined with active-passive joint control, it can leverage the advantages of both active and passive constraint layer damping, leading to effective vibration reduction in structures such as beams, plates, and shells [1,2]. The piezoelectric layer is commonly employed for active control due to its remarkable advantages. To mitigate vibration, an active control system is integrated, utilizing both positive and negative piezoelectricity. While certain passive constrained damping layers (PCLD) have limitations, an active constraint layer can enhance the shear deformation of the viscoelastic layer [3]. Due to its excellent mechanical properties and cost-effectiveness, the active constraint layer has been widely utilized in real-world engineering applications. In the event of active control mechanism failure or damage, a passive damping layer, incorporating viscoelastic materials, can convert vibration energy into thermal energy dissipation [4], thereby ensuring system safety and stability. The field has garnered increasing attention from scholars [5,6,7]. 

Creating an accurate model is crucial for analyzing the structure of ACLD and implementing vibration control. Using piezoelectric materials as brakes, Baz et al. [8] introduced an ACLD-treated beam model in 1988. They contrasted it with conventional PCLD-treated beams and outlined its benefits. In 2001, SHI et al. [9] combined the finite element method with the GHM model to complete the modeling of the ACLD structure and used an LQG controller to control the vibration, thus opening the door for later researchers to examine active control. ACLD beams were employed as the research subject by Li et al. [10] who used simulations and experiments to examine the effects of various control signals on vibration reduction. A sandwich beam finite element with 18 physical nodes of mechanical and potential degrees of freedom was proposed by A. R. Damanpack et al. [11,12]. Huang et al. [13,14] proposed two finite element models of viscoelastic sandwich structures based on shear and compression deformation theories. Gao et al. [15] proposed a finite element formula using the Z-shaped assumption to improve the accuracy of the sandwich intelligent structure model. Due to the high tracking precision required by the control system in this article, an accurate model must be established in order to solve the issue of high degrees of freedom in conventional finite element methods. 

Furthermore, some academics have begun utilizing innovative techniques to optimize the control parameters of ACLD structures, building upon conventional active control methods. Mojtaba Biglar et al. [16] found the influence of piezoelectric plate position on vibration reduction based on a genetic algorithm for piezoelectric plate structures. After optimizing the control weighting parameters using particle swarm optimization, Huang et al. [17] applied the optimized parameters in their control strategy. The results they achieved were significant. Henan Song et al. [18] proposed a NARX neural network to identify the dynamic model of a cantilever beam. Tian et al. [19] used a genetic algorithm to optimize linear–quadratic regulators for piezoelectric composite laminated beams. Osama Abdeljaber et al. [20] proposed an optimization method using artificial neural networks to reduce the vibration of flexible cantilever plates. Chai et al. [21] indicate that linear–quadratic–Gaussian control has a better vibration reduction effect on laminated plates. Liu et al. [22] proposed an optimization method for composite laminates based on artificial neural networks and genetic algorithms. Zhang et al. [23] combined feedback/feedforward linear–quadratic–Gaussian control to reduce the vibration of the ACLD board. Saber Yaghoobi et al. [24] compared linear–quadratic–Gaussian control and PID control in the design of an active controller. Abhishek S [25] and others have studied the influence of adaptive linear–quadratic–Gaussian control on vibration control, and the results show that the LQG controller is more suitable for suppressing vibration and noise of structures in a random excitation environment. Li et al. [26] used a differential evolution algorithm to optimize the instantaneous disturbance of LQR control of a piezoelectric flexible beam and compared the simulation and experimental data. Tarcio M.P. Silva et al. [27] used particle swarm optimization to optimize the positive feedback controller for vibration reduction of composite sandwich panels. 

Most existing optimization techniques focus on a particular objective, such as optimizing the control settings of a single structure to reduce vibration using neural network algorithms. They seldom address systems with multiple locations and structures. To the best of the author’s knowledge, vibration optimization of composite material structures with multiple objectives has not been conducted.

Based on first-order shear deformation theory and the finite element method, this paper develops corresponding optimization models and algorithms for the aforementioned problems in the MATLAB platform. We establish a structural model for viscoelastic sandwich beams and derive the governing equations using Hamilton’s principle. The simplified model undergoes LQR active control, and a Kalman filter is incorporated to form an LQG system, enabling simultaneous optimal control and state estimation. The genetic algorithm is employed to optimize the parameters Q and R based on this foundation. Three cantilever beam models with different coverage are utilized for vibration control. The use of the genetic algorithm has been found to significantly increase the efficiency of vibration control. Additionally, the control system incorporating a Kalman filter can play a valuable role in suppressing periodic interference signals. This paper evaluates the model’s applicability in industries such as aviation and the automobile industry. It compares the performance of active controller optimization with conventional control systems in terms of signal input vibration resistance across different settings. This research achievement has established a finite element model with higher accuracy and lower degree of freedom for the aerospace and automotive fields and provided an innovative and efficient vibration control scheme. At the same time, it also provides a useful reference for the application of active damping structure in theory and practice.

## 2. Finite Element Modeling

### 2.1. Basic Assumptions of the Model

(1) The foundation beam layer and piezoelectric constraint layer can be regarded as the Euler-Bernoulli beam.
(2) The layers are fully bonded without relative displacement.
(3) The material density of each layer is uniform, which conforms to the basic assumption of material mechanics.
(4) The influence of the moment of inertia of each layer is ignored in the analysis.
(5) Regardless of the compression deformation in the Z direction, the transverse displacement (deflection) ω of the three layers is the same.
(6) The viscoelastic damping coefficient is only discussed in viscoelastic linear theory.
(7) The piezoelectric force applied on the damping sandwich beam is uniformly distributed in the element.

### 2.2. Geometric Deformation Relationships and Finite Element Elements

**Figure 1:**
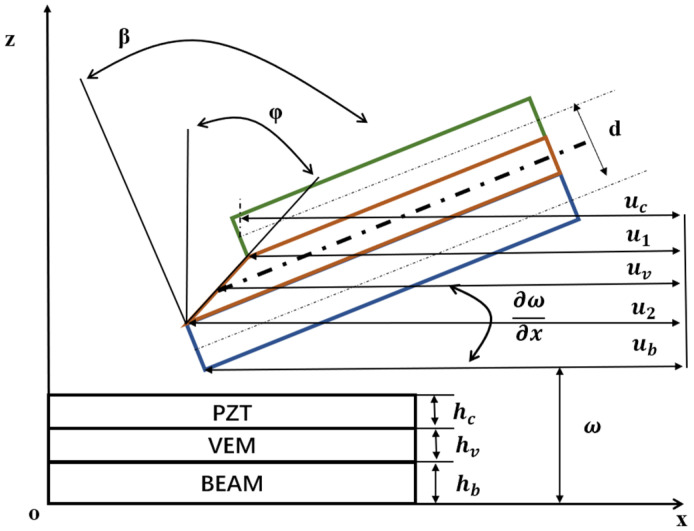
Geometric deformation diagram of viscoelastic sandwich beams.

According to the first-order shear deformation theory, the geometric deformation relationship of viscoelastic sandwich beam structure is shown in Figure 1, Where $\frac{\partial \omega }{\partial x}$ is the cross-section rotation of ACLD beam; φ is the shear angle of Viscoelastic layer; is the shear strain of viscoelastic layer; *u_c_*, *u_b_* are the transverse displacement in PZT layer and base beam layer. *h_c_*, *h_b_*, and *h_v_* is the thickness of the piezoelectric layer, base beam layer, and viscoelastic layer, respectively.

The finite element used in this article is a 2-node 8-degree-of-freedom beam element, and the displacement vector of the element is:

(1) $$\{V^{e}\}={\left\{w_{i}\;\theta_{i}\;u_{bi}\;u_{ci}\;w_{j}\;\theta_{j}\;u_{bj}\;u_{cj}\right\}}^{T}$$

### 2.3. Kinetic Equation

According to the Hamilton principle, the variational equation can be obtained:

(2) $$\int_{t1}^{t2}\delta (T-U)dt+\int_{t1}^{t2}\delta Wdt=0$$

The GHM model is introduced in this paper in viscoelastic materials, and the GHM model characterizes the damping properties of viscoelastic materials by introducing dissipative coordinates. These dissipation coordinates are introduced into the mass, stiffness, and damping matrices and are used to accurately describe the dissipative behavior of the material. The composite Shear modulus of viscoelastic materials is expressed by the GHM model using the micro vibrator model [28], which $s\tilde{G}(s)$ represents the complex Shear modulus function of materials:

(3) $$s\tilde{G}(s)=G^{\infty }[1+\sum_{k=1}^{N}\alpha_{k}\frac{s^{2}+2{\hat{\xi }}_{k}{\hat{\omega }}_{k}s}{s^{2}+2{\hat{\xi }}_{k}{\hat{\omega }}_{k}s+{{\hat{\omega }}_{k}}^{2}}]$$

(4) $${\hat{Z}}_{k}(s)=\frac{{\hat{\omega }}_{k}}{s^{2}+2{\hat{\xi }}_{k}{\hat{\omega }}_{k}s+{{\hat{\omega }}_{k}}^{2}}X(s)$$

Substitute the total kinetic energy *T* and total potential energy *U* of the system into the Equation (2), and then perform inverse Laplace transform to organize the dynamic model of the viscoelastic sandwich beam. The expression in the time domain is [13]:

(5) $$\tilde{M}\ddot{q}+\tilde{D}\dot{q}+\tilde{K}q=\tilde{f}$$

where

(6) $$\tilde{M}=\left[\begin{array}{cccc} M^{e} & 0 & \cdots & 0\\ 0 & \frac{\alpha_{1}}{{\hat{\omega }}_{1}^{2}}\Delta & 0 & \vdots \\ \vdots & 0 & \ddots & 0\\ 0 & \cdots & 0 & \frac{\alpha_{N}}{{\hat{\omega }}_{N}^{2}}\Delta \end{array}\right],\tilde{D}=\left[\begin{array}{cccc} 0 & 0 & \cdots & 0\\ 0 & 2{\hat{\xi }}_{1}\frac{\alpha_{1}}{{\hat{\omega }}_{1}}\Delta & 0 & \vdots \\ \vdots & 0 & \ddots & 0\\ 0 & \cdots & 0 & 2{\hat{\xi }}_{N}\frac{\alpha_{N}}{{\hat{\omega }}_{N}}\Delta \end{array}\right]$$

(7) $$\tilde{K}=\left[\begin{array}{cccc} K_{e}+\tilde{k}(1+\sum_{K=1}^{N}\alpha_{k}) & -\alpha_{1}R & \cdots & -\alpha_{N}R\\ -\alpha_{1}R^{T} & \alpha_{1}\Delta & 0 & 0\\ \vdots & 0 & \ddots & 0\\ -\alpha_{N}R^{T} & 0 & 0 & \alpha_{N}\Delta \end{array}\right],q=\left\{\begin{array}{c} x\\ Z_{1}\\ \vdots \\ Z_{N} \end{array}\right\},\tilde{f}=\left\{\begin{array}{c} f_{e}\\ 0\\ 0\\ 0 \end{array}\right\}$$

where: $\tilde{k}=G^{\infty }K_{v}^{e},K_{v}^{e}=R_{v}\Delta_{v}R_{v}^{T},\Delta_{v}$ is the diagonal matrix composed of positive eigenvalues of viscoelastic layer $K_{v}^{e}$, *R_v_* is the eigenvector matrix composed of the corresponding eigenvectors of $K_{v}^{e}$ as columns, $\Delta =G^{\infty }K_{v}^{e},R=R_{v}\Delta_{v},R=R_{v}\Delta ,Z_{j}=R_{v}^{T}{\hat{Z}}_{j}$ (*j* = 1, 2, …, *N*).

The dynamic finite element equation of viscoelastic sandwich beams can be obtained by assembling each element as follows:

(8) $$M\ddot{q}+D\dot{q}+Kq=f$$

### 2.4. Model Downgrading

It is crucial to lower the order of the system since the finite element model’s huge dimensions make active control difficult.

#### 2.4.1. Dynamic Condensation in Physical Space

The z-direction displacement of the constraint layer’s line displacement in the x and y directions in the structure is taken as the main degree of freedom, and other physical and dissipative degrees of freedom are taken as the secondary degrees of freedom. After iterative condensation, the coordinates that have less impact on the system can be omitted [29], as shown in the Equation (9):

(9) $$\left[\begin{array}{cc} M_{mm} & M_{ms}\\ M_{sm} & M_{ss} \end{array}\right]\left\{\begin{array}{c} {\ddot{X}}_{m}(s)\\ {\ddot{X}}_{s}(s) \end{array}\right\}+\left[\begin{array}{cc} D_{mm} & D_{ms}\\ D_{sm} & D_{ss} \end{array}\right]\left\{\begin{array}{c} {\dot{X}}_{m}(s)\\ {\dot{X}}_{s}(s) \end{array}\right\}+\left[\begin{array}{cc} K_{mm} & K_{ms}\\ K_{sm} & K_{ss} \end{array}\right]\left\{\begin{array}{c} X_{m}(s)\\ X_{s}(s) \end{array}\right\}=\left\{\begin{array}{c} F_{m}(s)\\ F_{s}(s) \end{array}\right\}$$

The kinetic equation after condensation is:

(10) $$M_{E}^{\left(i\right)}{\ddot{X}}_{m}+D_{E}^{\left(i\right)}{\dot{X}}_{m}+K_{E}^{\left(i\right)}X_{m}=F_{E}^{\left(i\right)}$$

#### 2.4.2. Modal Decoupling in State Space

Introducing the auxiliary equation $M_{E}\dot{X}-M_{E}\dot{X}=\left[0\right]$ in the Equation (10), the viscoelastic sandwich structure system model can be represented in state space as:

(11) $$\left\{\begin{array}{c} \ddot{X}\\ \dot{X} \end{array}\right\}={\left[\begin{array}{cc} 0 & M_{E}\\ M_{E} & D_{E} \end{array}\right]}^{-1}\left[\begin{array}{cc} M_{E} & 0\\ 0 & -K_{E} \end{array}\right]\left\{\begin{array}{c} \dot{X}\\ X \end{array}\right\}+{\left[\begin{array}{cc} 0 & M_{E}\\ M_{E} & D_{E} \end{array}\right]}^{-1}\left\{\begin{array}{c} F_{E}\\ 0 \end{array}\right\}+{\left[\begin{array}{cc} 0 & M_{E}\\ M_{E} & D_{E} \end{array}\right]}^{-1}\left\{\begin{array}{c} F_{E}\\ 0 \end{array}\right\}$$

The equation can be written as Equation of state:

(12) $$\left\{ \begin{array}{l} \dot{Y}=AY+Bf\\ Z=CY \end{array}\right.$$

where:

(13) $$A=\left[\begin{array}{cc} -M_{E}^{-1}D_{E} & -M_{E}^{-1}K_{E}\\ 0 & I \end{array}\right],B=\left[\begin{array}{c} M_{E}^{-1}F_{E}\\ 0 \end{array}\right],Y=\left[\begin{array}{c} \dot{X}\\ X \end{array}\right]$$

where *A* is the system dynamics matrix of the structure, *B* is the piezoelectric control force matrix, *C* is the sensor output matrix, and f is the external excitation received by the system.

This paper retains the system’s first three modes after modal reduction. It is a lower-order mode with fewer components of higher-order natural modes since vibration is mostly caused by low-frequency vibrations. Only the first three modes are taken into consideration when choosing the order of active vibration control since the low-frequency elastic mode is the primary mode that significantly influences the features of the system.

## 3. Active Control

### 3.1. Linear–Quadratic–Regulator Control

The main objective of this optimal control strategy is to minimize the quadratic objective function J of the intended state feedback controller [30]; the gain K is determined solely by the weighting matrices Q and R. The parameters of Q and R need to be established as they directly affect the effectiveness of the control.

Where the state space linear Time-invariant system equation is:

(14) $$\dot{x}(t)=Ax(t)+Bu(t)$$

*K* is the state feedback gain matrix, which can obtain the closed-loop system matrix:

(15) $$\dot{x}(t)=(A-BG)x(t)$$

The expression form of the commonly used objective function *J* is:

(16) $$J=\int_{0}^{\infty }(x^{T}(t)Qx(x)+u^{T}(t)Ru(t))dt$$

### 3.2. Kalman Filter and Linear–Quadratic–Gaussian Control

This study combines the Kalman filter and LQR control to implement linear–quadratic–Gaussian (LQG) control, which effectively mitigates the impact of external disturbances on the system [31]. Upon introducing actual noise, the system’s state equation can be expressed as follows:

(17) $$\left\{ \begin{array}{l} \dot{x}(t)=Ax(t)+Bu(t)+w\\ y(t)=Cx(t)+v \end{array}\right.$$

The LQG regulator has the following state space equation:

(18) $$\frac{d}{dt}\tilde{x}=[A-LC-(B-LD)K]\tilde{x}+Ly$$

where *w* and *v* are white noise, The Kalman gain *L* is determined by the algebraic Riccati equation.

**Figure 2:**
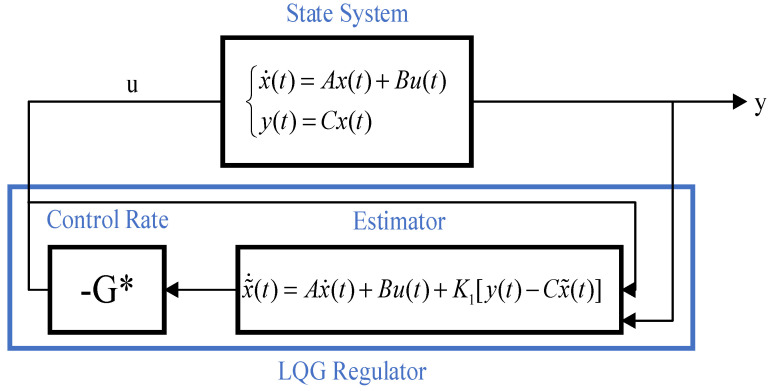
LQG frame diagram.

The linear quadratic form Gaussian controller calculates the control input while the Kalman filter estimates the system state. Figure 2 displays the block diagram of its structure. The system’s behavior can be predicted more precisely using linear–quadratic–Gaussian control, which can then apply the appropriate control measures. Linear–quadratic–Gaussian control is, hence, typically utilized in control applications that demand more accuracy and robustness.

### 3.3. Genetic Algorithm Optimization Control Parameter Model

**Figure 3:**
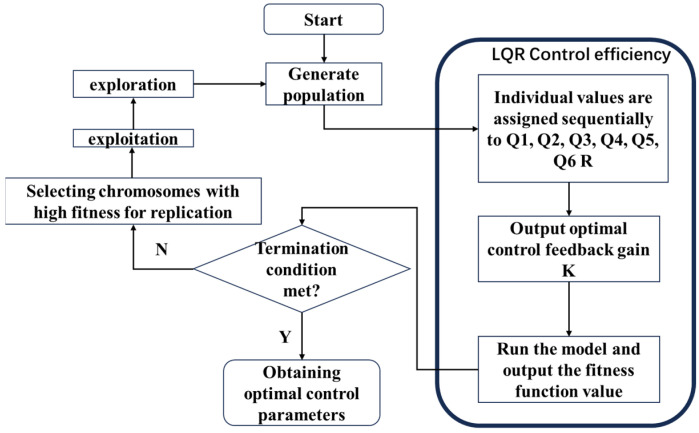
Flow chart of the GA algorithm optimizes Q R parameters.

It is impossible to separate the optimization of the control parameters Q and R from LQR control, also known as linear–quadratic–Gaussian control. In this study, iterative evolution is used to gradually reach the best answer, while a genetic algorithm is used to mimic the natural evolution process [32]. Three processes, selection, crossover, and mutation, help the genetic algorithm keep improving by favoring fitter individuals and gradually approaching the optimal result. The exact evolutionary method flowchart for the evolution of the parameters Q and R is shown in Figure 3.

In this paper, we simulate the process of superiority and inferiority in biological evolution and continuously optimize the iterative process of individuals in the population to the optimal genes to determine the optimal control parameters. The weighting matrices Q and R of the parameters to be optimized are:

(19) $$\begin{array}{c} Q=diag[x(i,1),x(i,2),,x(i,6)]\\ R=x(i) \end{array}$$

The LQR cost function to be optimized is:

(20) $$J(U)=\overset{N-1}{\underset{n=1}{\Sigma }}\left(X_{n}^{T}QX_{n}+u_{n}^{T}Ru_{n}\right)+x_{N}^{T}Q_{z}x_{N}$$

where the function parameters $U=(u_{0},u_{1},\ldots ,u_{N})$, *Q*, *Qz*, and *R*, are positive definite matrices, *N* is a time horizon, $x_{N}^{T}Q_{z}x_{N}$ is a measure of end-state bias, $X_{n}^{T}QX_{n}$ is a measure of state bias, $u_{n}^{T}Ru_{n}$ is a measure of input size.

## 4. Numerical Validation and Analysis

### 4.1. LQR Parameter Selection

To study the optimal vibration control scheme, this section first selects the appropriate control parameters by analyzing five groups of control parameters and comparing their control efficiency in time domain space and frequency domain space. After determining the specific parameters and structure, vibration analysis will be performed on the system of actively constrained damped cantilever beam structure under different excitations using LQR control. The effect of different parameter variations on the free vibration decay curves of the system was investigated by applying initial displacements to the structure. 

This section addresses the pairwise forward third-order modal vibration patterns of an actively constrained layer-damped cantilever beam structure. The finite element model is equally divided into 7 cells, and the simulation is structured as a viscoelastic and piezoelectric layer covering the first 4 cells, with the boundary condition of one end solidly supported and one end free. Set the length L of the base beam to 0.35 m and the width to 0.015 m.

**Figure 4:**
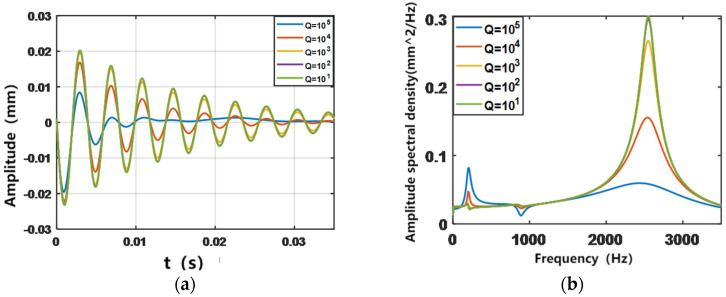
The influence of Q-matrix variation on LQR control in time and frequency domains. (**a**) Time domain; (**b**) Frequency domain.

**Figure 5:**
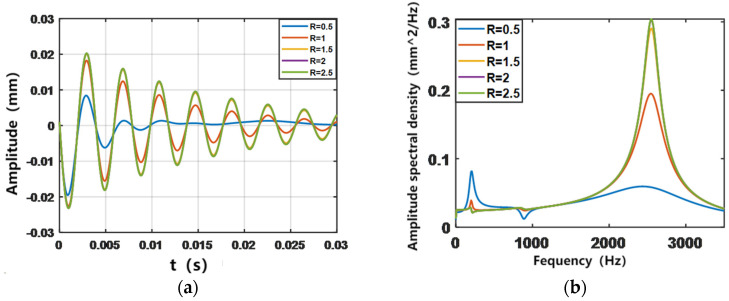
The influence of R variation on LQR control in time and frequency domains. (**a**) Time domain; (**b**) Frequency domain.

To determine the appropriate control parameters and the range of application, in this section, the parameters Q and R of LQR control are studied in groups and five sets of data are selected for analysis as Q_1_ = I × 10^1^, Q_2_ = I × 10^2^, Q_3_ = I × 10^3^, Q_4_ = I × 10^4^, Q_5_ = I × 10^5^ and R_1_ = 0.5, R_2_ = 1, R_3_ = 1.5, R_4_ = 2, and R_5_ = 2.5. The output simulation is the amplitude response of the ACLD cantilever beam. Figure 4 and Figure 5 show, respectively, the simulation results of the vibration control response of the structure with different active control parameters in the time and frequency domains. From the frequency-domain diagram, it can be clearly observed that the vibration is mainly provided by the third-order vibration mode of the structure, and the contribution of the first two orders is less; in the time-domain diagram, it can be seen that the gap between the vibration suppression effect of different control parameters on the structure is very obvious. Among them, the set of control parameters Q = I × 10^5^ and R = 0.5 can effectively limit the structural vibration. 

### 4.2. Model Validation

This chapter presents multiple numerical examples that demonstrate the accuracy of the finite element model for the active damping beam in the subsequent vibration analysis. It also assesses the stability and effectiveness of the optimization control parameters using a genetic algorithm and the subsequent linear–quadratic–Gaussian control. The structural variables are presented in Table 1.

**Figure 6:**
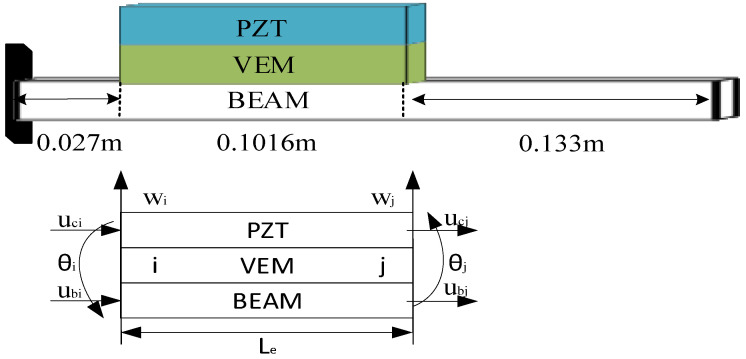
Finite element model of a cantilever beam.

In this work, the cantilever beam model partially covered with ACLD is divided into ACLD cells and ordinary beam cells, with two ordinary beam cells on the left side, two ACLD cells in the center, and three ordinary beam cells on the right side, for a total of seven finite element cells. A 2-node 8-degree-of-freedom finite element model [9] was used for the ACLD cell to validate the finite element method for the viscoelastic sandwich beam structure under the solidly supported-freedom boundary conditions, and vibration optimization analyses were performed in conjunction with the intrinsic frequency data of the cantilever beam of the ACLD cell. The GHM model parameter is G^∞^ = 5 × $10^{5}$ Pa, α = 6, ς = 4, ω = 10,000 rad/s, as shown in Figure 6.

Table 2 presents the first four natural frequencies of the active damping cantilever beam, calculated using the finite element method in this paper and compared with data from the literature. The method in this paper exhibits an error range of 0.25% to 3.21%, indicating the high accuracy of the finite element model employed.

### 4.3. Model Reduction

**Figure 7:**
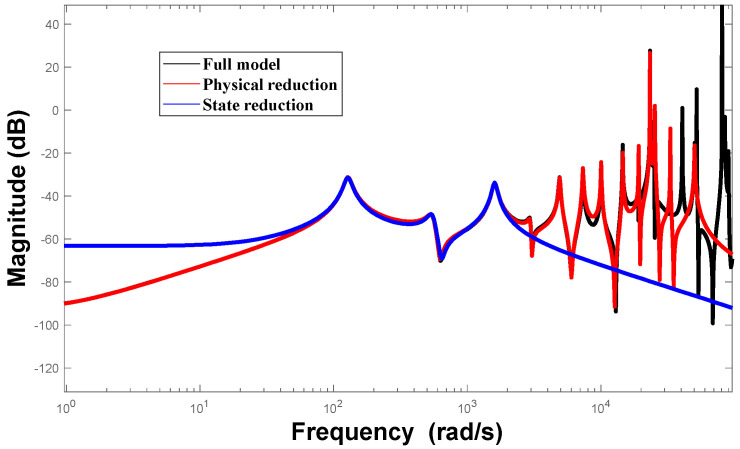
Comparison before and after model reduction.

Figure 7 illustrates the preserved frequencies of the model after both physical space reduction and state space reduction. It is evident that the frequencies of the model, before and after order reduction, remain accurate, retaining the first three main modes and satisfying the observability and controllability requirements of the control system. Each mode represents an independent real number mode that can be directly utilized in the design of active controllers.

### 4.4. Vibration Analysis

#### 4.4.1. Vibration Analysis with Different Coverage

**Figure 8:**
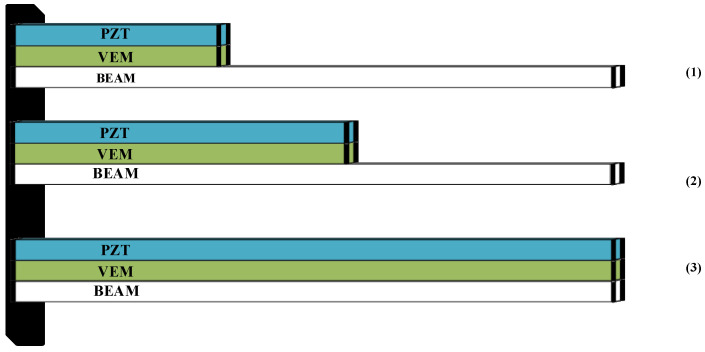
Cantilever beams with different structures.

This paper investigates the free vibration control of viscoelastic sandwich beams, as depicted in Figure 8. The specific structure consists of three sections: Structure 1 with a coverage rate of 2/7 L, Structure 2 with a coverage rate of 4/7 L, and Structure 3 fully covered. The study examines vibration attenuation under both uncontrolled and controlled conditions. An initial displacement is applied to the structure, and the output is measured as the lateral displacement response at the free end of the cantilever beam. The length of the cantilever beam is adjusted to 0.35 m, and the width is set to 0.15 m. All other parameters remain consistent with Table 1.

Table 3 demonstrates that as the coverage of ACLD patches increases, the natural frequency of the system decreases. This is due to the unchanged stiffness of the cantilever beam, while the mass and damping increase, resulting in a lower natural frequency [5].

#### 4.4.2. Comparison of LQR and LQG Control Effects 

**Figure 9:**
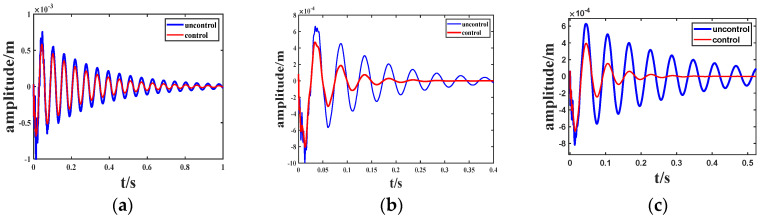
Amplitude of different structures. (**a**) Structure 1; (**b**) Structure 2; (**c**) Structure 3.

Figure 9 presents the amplitude attenuation diagram of LQR control for structures (1), (2), and (3) with parameter values Q = I × 10^5^ and R = 0.5. It is evident that structure (2) exhibits the most prominent active control effect, with a damping effect of 18.2% and a vibration attenuation time of 0.2 s. For structure (2), this article will employ the LQG control method to further enhance control and optimization.

**Figure 10:**
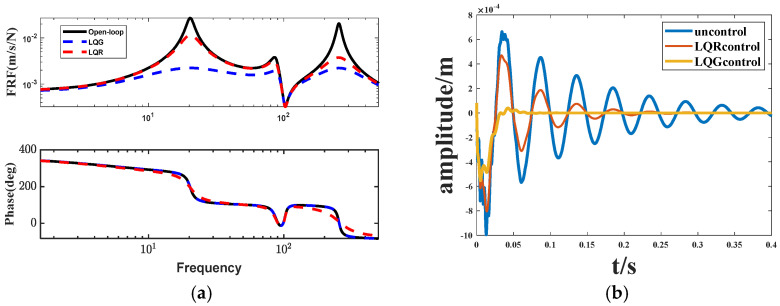
LQG control. (**a**) Bode diagram; (**b**) Amplitude diagram.

From Figure 10, we can see that the structure (2) LQG control relative to the LQR and open-loop system on the vibration attenuation has a better control effect and tracking accuracy, LQG control relative to the LQR control on the vibration attenuation time is shortened by 0.15 s control effect is obvious. 

**Figure 11:**
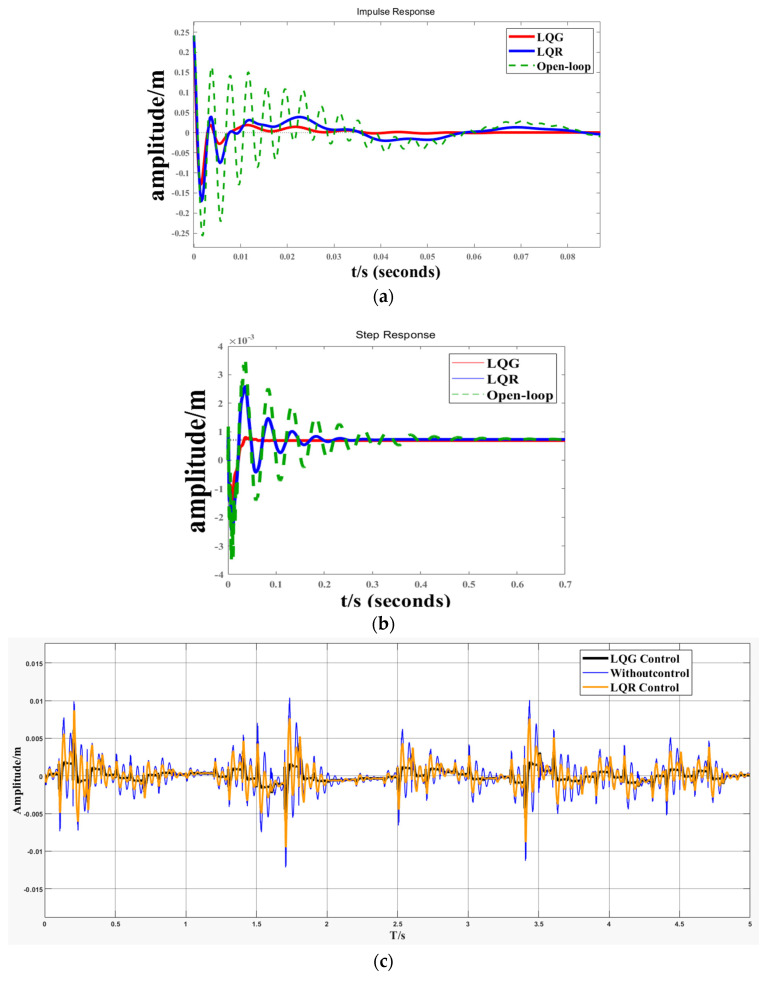
Response under different signal inputs. (**a**) Impulse Response; (**b**) Step Response; (**c**) Gaussian White Noise Response.

Figure 11 illustrates the vibration responses of the LQR control and linear–quadratic–Gaussian (LQG) control applied to the structure 2 system with various signal inputs. It is evident from (b) and (c) that the addition of a Kalman filter enhances the damping effect of the LQG control on periodic signals. This improvement indicates an increased stability margin, enhanced system stability, and suppressed amplitude and frequency of vibration. At the same time, the impact on pulse signals is relatively minimal.

### 4.5. Genetic Algorithm Optimization of Control Parameters

**Figure 12:**
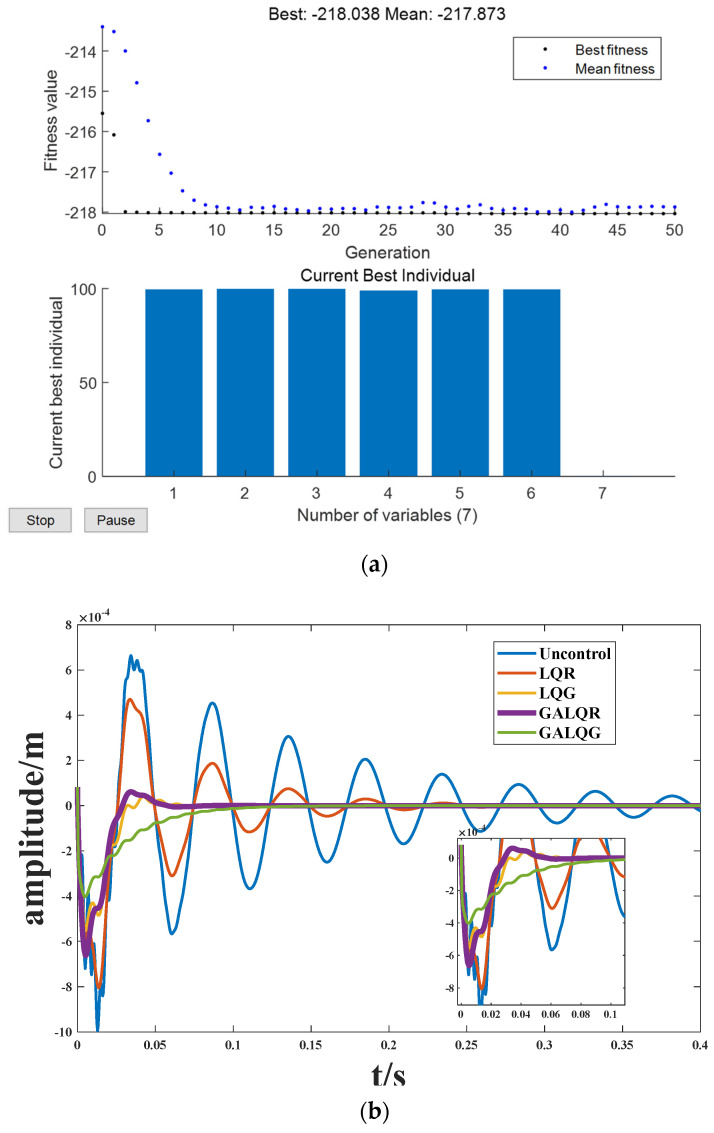
Genetic algorithm optimization. (**a**) Optimization process; (**b**) Amplitude diagram.

Genetic algorithm is a powerful and widely used stochastic search and optimization technique based on the principles of natural selection and genetics. The GA algorithm in this study employs integer coding to find the optimal Q and R using a single-point crossover operation. Utilizing the GA algorithm, the linear–quadratic–Gaussian (LQG) control parameters are enhanced, and specific data analysis is conducted for structure 2. The initial chromosomes are randomly selected based on the population size, and the optimal control parameters are eventually found by iterating over the optimal genes. Where population density = 200; number of iterations = 50; fitness = 1000; mutation rate is 0.05; and the range to be optimized is 0.1 to 100. Figure 12a demonstrates the discovery of the best Q and R values after 50 generations of the genetic algorithm. Over 10 iterations, the fitness values of the objective function gradually converge. Figure 12b clearly indicates the substantial improvement in the effectiveness of active vibration control achieved through parameter optimization using a genetic algorithm.

## 5. Conclusions

This paper presents a combined finite element and GHM model to create the ACLD cantilever beam model. The model is subsequently reduced using physical and state approaches. To enhance vibration control, a Kalman filter is incorporated into the linear–quadratic regulator, resulting in the linear–quadratic–Gaussian control. Additionally, a genetic algorithm is employed to optimize the Q and R parameters. The effect of vibration control is examined by analyzing beam structures with different ACLD patch coverages. The obtained numerical results lead to the following conclusions:

1. The combined finite element and GHM model for the cantilever beam demonstrates improved accuracy and reduced degrees of freedom compared to the existing literature. The joint reduction of physical and state spaces proves accurate and effective, particularly in preserving the initial modal characteristics. This reduced-order processing is useful in the aerospace and automotive fields to reduce the computational burden in the design of complex systems while ensuring model accuracy. For instance, the structural vibration control of an aircraft wing or an automobile body can be optimized with the help of this method to ensure high stability and safety even at high speeds.
2. Increasing the ACLD patch coverage improves the passive seismic performance of the cantilever beam. However, in active control, full coverage may not always be the optimal choice. When compared to active control, a 4/7 L coverage rate for structure (2) results in a 0.05 s reduction in control time compared to full coverage. In reality, especially in the seismic design of bridges and high-rise buildings, this finding informs the economy of the control system, reducing materials while still achieving the desired control.
3. By utilizing the Kalman filter, the LQG controller can significantly reduce noise and interference, leading to improved response speed and accuracy of the control system. Compared to traditional LQR control, the maximum amplitude can be reduced by 31.1%. Additionally, the LQG controller effectively suppresses periodic signals like Gaussian white noise, indicating that linear–quadratic–Gaussian control is more efficient for structures exposed to random excitation environments. However, the effect on single pulse signals is not as noticeable.
4. By optimizing the weighted parameters of the controller through the genetic algorithm, the active control effect has significantly improved. The optimized parameters better meet the system’s control requirements and tracking accuracy. Compared to manual parameter tuning methods, genetic algorithms can more quickly find the optimal solution. This is important in areas such as drones and autonomous vehicles, where the requirements for real-time control systems are very high. Genetic Algorithms can quickly find the optimal control parameters to ensure that the system can still maintain stable control accuracy and response speed in complex environments.

This paper explores how dividing the ACLD cantilever beam system into active damping patches can improve the effectiveness of vibration reduction. Comparative analysis revealed that adopting appropriate active control strategies can effectively reduce the system’s response frequency. These research results offer an innovative and efficient vibration control scheme for the aerospace and automotive fields, as well as theoretical and practical references for the application of active damping structures.

## Figures and Tables

**Table 1:** Material parameters.

Parameter	L/m	W/m	THK/m	ρ/kg/m^3^	E /Gpa	PR	d31/m/V
Base layer	0.2616	0.0127	0.002286	7600	7.4 × $10^{10}$	0.3	
PZT layer	0.1016	0.1027	0.000762	7600	6.67 × $10^{10}$	0.3	1.75 × $10^{10}$
VEM layer	0.1016	0.1027	0.00025	1250		0.3	

**Table 2:** Natural frequency comparison.

Modal	Frequency/Hz (Paper) [9]	Frequency/Hz (Present)	Error
1 mode	27.90	27.83	0.25%
2 mode	150.12	147.83	1.52%
3 mode	442.97	429.66	3.00%
4 mode	831.76	805.08	3.21%

**Table 3:** The first four natural frequencies of different structures.

Natural Frequencies	Structure 1	Structure 2	Structure 3
1	17.02	19.85	16.37
2	97.45	83.23	82.52
3	247.04	235.25	211.43
4	806.16	725.19	690.46

## Data Availability

Data sharing is not applicable.
